# Dehydroeburicoic Acid, a Dual Inhibitor against Oxidative Stress in Alcoholic Liver Disease

**DOI:** 10.3390/ph16010014

**Published:** 2022-12-22

**Authors:** Shasha Cheng, Yi Kuang, Guodong Li, Jia Wu, Chung-Nga Ko, Wanhe Wang, Dik-Lung Ma, Min Ye, Chung-Hang Leung

**Affiliations:** 1 State Key Laboratory of Quality Research in Chinese Medicine, Institute of Chinese Medical Sciences, University of Macau, Macau SAR 999078, China; 2 State Key Laboratory of Natural and Biomimetic Drugs, School of Pharmaceutical Sciences, Peking University, Beijing 100191, China; 3 Zhuhai UM Science and Technology Research Institute, Zhuhai 519031, China; 4 Department of Chemistry, Hong Kong Baptist University, Hong Kong SAR 999077, China; 5 Institute of Medical Research, Northwestern Polytechnical University, Xi’an 710072, China; 6 Department of Biomedical Sciences, Faculty of Health Sciences, University of Macau, Macau SAR 999078, China

**Keywords:** alcoholic liver disease (ALD), Keap1–Nrf2 protein–protein interaction (PPI), glycogen synthase kinase 3β (GSK3β), hepatoprotective

## Abstract

Alcoholic liver disease (ALD) is a complicated disease which can lead to hepatocellular carcinoma; however, there is a lack of satisfactory therapeutics. Dehydroeburicoic acid (DEA) (**1**), a triterpenoid isolated from *Antrodia cinnamomea*, has been reported to act against ALD, but its mechanisms of action are still not clear. In this study, we report for the first time the use of DEA (**1**) as a dual inhibitor of the Keap1–Nrf2 protein–protein interaction (PPI) and GSK3β in an in vitro ALD cell model. DEA (**1**) engages Keap1 to disrupt the Keap1–Nrf2 PPI and inhibits GSK3β to restore Nrf2 activity in a Keap1-independent fashion. DEA (**1**) promotes Nrf2 nuclear translocation to activate downstream antioxidant genes. Importantly, DEA (**1**) restores the mitochondrial dysfunction induced by ethanol and generates antioxidant activity in the ALD cell model with minimal toxicity. We anticipate that DEA (**1**) could be a potential scaffold for the further development of clinical agents for treating ALD.

## 1. Introduction

Excessive alcohol consumption leads to oxidative stress in the liver as a result of alcohol metabolism, resulting in inflammatory damage and the injury of liver cells [1]. Almost 20% of alcoholics are eventually diagnosed with alcoholic liver disease (ALD) [2]. The most common therapeutic options for ALD can be divided into opioid receptor antagonists, supplements to modulate liver metabolism, and therapeutics that regulate alcohol metabolism [3]. However, these treatments have been associated with side effects including dizziness, drug dependence, dermatitis, vomiting, and leukopenia [4]. Thus, there are currently no highly satisfactory therapeutic options for ALD, and hence there is an unmet clinical need to develop more effective and safer drugs for patients with ALD.

Increasing evidence indicates that alcohol can damage mitochondria and destroy cellular homeostasis in the liver [5,6,7]. One mechanism that cells use to defend against alcohol–induced injury is through the activation of nuclear respiratory factor-2 (Nrf-2). Nrf2 orchestrates a complex, self-protective antioxidant response involving numerous signaling axes [8]. Upregulating Nrf2 has been envisioned as an effective tool for combating alcohol-induced acute liver injury [9,10]. Normally, the level of Nrf2 in the cytosol is low due to ubiquitination and proteasomal degradation regulated by a Kelch-like ECH-associated protein (Keap1) [11,12]. However, the presence of oxidants can oxidize cysteine residues in Keap1 into disulfides or conjugate them to electrophiles, resulting in the nuclear translocation of Nrf2 and the activation of antioxidant genes [13], including heme oxygenase-1 (*HO*–*1*), NAD(P)H dehydrogenase [quinone] 1 (*NQO1*), and the mitochondrial superoxide dismutase 2 (*SOD2*) [14,15]. In addition, Nrf2 activation increases the expression levels of nuclear respiratory factor-1 (Nrf-1) and peroxisome proliferator-activated receptor γ coactivator 1α (PGC1α), which in turn modulates the expression of mitochondrial respiratory subunits and translational components [16]. The genes activated by the antioxidant responsive element (ARE) protect cells against any damage caused by reactive oxygen species (ROS) [17]. In addition to Keap1, glycogen synthase kinase 3β (GSK3β) is another negative regulator of Nrf2 that acts to downregulate the antioxidant stress response [18,19]. GSK3β promotes the nuclear exclusion and degradation of Nrf2 in a Keap1-independent fashion in stressed or injured cells with active Nrf2, thereby reducing the degree of protection conferred by Nrf2 activity [20,21]. Therefore, increasing the Nrf2 antioxidant activity via targeting Keap1 or GSK3β could be a potential approach for treating ALD [22].

Most pharmacological approaches towards Nrf2 activation are focused on the inhibition of Keap1 [23,24]. Several Keap1 inhibitors have been reported in the last decade, and the vast majority of these covalently modify cysteine residues in Keap1 [25]. Covalently targeting cysteines in Keap1 may lack selectivity due to the presence of other reactive cysteine residues in the cell, resulting in adverse side effects [26,27]. However, because cysteine residues are abundant in cells, the safety and specificity of covalent drugs are a concern [28]. Recently, protein–protein interaction (PPI) inhibitors of Keap1 and Nrf2, developed based on the X-ray structure of Keap1, have emerged as a new class of Nrf2 activators [29,30,31]. Our group has previously reported a cyclometalated iridium(III) metal complex as a Keap1–Nrf2 PPI inhibitor, which is a promising therapeutic agent for acetaminophen–induced acute liver injury [29,30]. However, no Keap1–Nrf2 inhibitor has yet entered the clinic for the treatment of human diseases [32,33]. Meanwhile, although a few GSK3β inhibitors have been reported for liver injury treatment, no GSK3β inhibitor to date has made it to the market [34].

*Antrodia cinnamomea*, a basidiomycete that is endemic to Taiwan, is an edible fungus that is a component of many traditional herbal medicines [35,36]. Either crude extracts or molecules isolated from *A. cinnamomea* have shown diverse biological properties, including antioxidant, anti-tumour, and anti-inflammatory activities [37,38]. Recently, research has shown that dehydroeburicoic acid (DEA) (**1**) from *A. cinnamomea* could inhibit alcoholic fatty liver disease (AFLD) by upregulating aldehyde dehydrogenase 2 family member (ALDH2) activity [39,40]. Additionally, DEA (**1**) also exhibited protective effects against non-alcoholic fatty liver disease (NAFLD) through activating ALDH2 and accelerating the elimination of ROS and harmful aldehydes [40]. Therefore, DEA (**1**) has the potential ability to treat ALD. However, its mechanisms of hepatoprotection in ALD are still not clear. Besides DEA (**1**), other triterpenoids have also been studied as GSK3β inhibitors and activators of Nrf2 because of their anti-inflammatory and antioxidant activities [41,42,43,44,45].

Research has shown that single–molecule drugs with two different biological activities may avoid some side effects and improve efficacy [46]. In this study, we discovered a potent dual Keap1–Nrf2 PPI and GSK3β inhibitor, DEA (**1**), and investigated its underlying mechanisms of action. Through in vitro fluorescence polarization (FP) screening, we identified DEA (**1**) as the most potent candidate for disrupting the Keap1–Nrf2 PPI (EC_50_ = 14.1 µM) from thirteen analogues isolated from *A. cinnamomea*. Moreover, DEA (**1**) inhibited GSK3β kinase activity with an EC_50_ value of 8.0 µM. In cells, low-toxicity compound DEA (**1**) engaged Keap1 and GSK3β to promote Nrf2 accumulation in the nucleus, leading to the upregulation of ARE transcriptional activity and increasing the expression of downstream antioxidant factors with more potent than the reported Keap1–Nrf2 PPI inhibitor ML334 in ALD model cells.

## 2. Results

### 2.1. DEA (**1**) Inhibits the Keap1–Nrf2 PPI and GSK3β Activity

**Figure 1:**
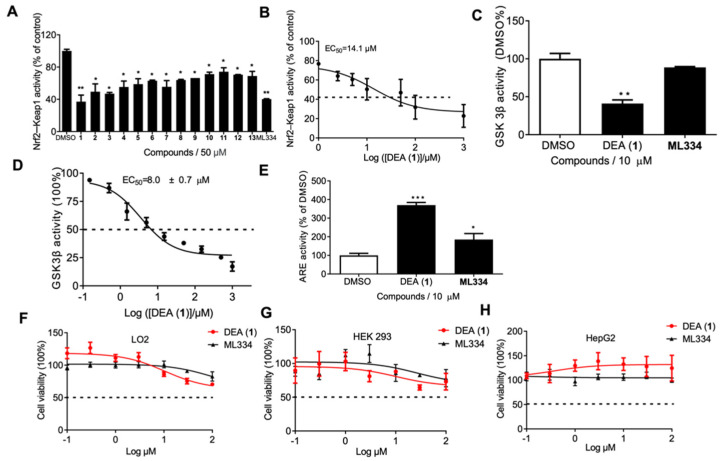
Screening of compounds **1**–**13** and identification of DEA (**1**) as a potent Keap1–Nrf2 inhibitor in vitro with low toxicity. (**A**) The binding activity of Keap1–Nrf2 was measured via an FP assay with the compounds added at 50 µM. (**B**) Dose-response effect of compound DEA (**1**) on Keap1–Nrf2 binding. (**C**) Compound DEA (**1**) inhibited GSK3β activity in vitro. (**D**) Dose-response effect of compound DEA (**1**) on GSK3β activity. (**E**) Compound DEA (**1**) enhances ARE activity in LO2 cells determined by the dual luciferase assay. (**F**–**H**) The cytotoxicity of DEA (**1**) and ML334 on LO2, HEK 293T, and HepG2 cell lines for 48 h. Cells were treated with 0–100 µM of DEA (**1**) and ML334 for 48 h and cytotoxicity was detected using the MTT assay. Data are represented as mean ± SD. * *p* < 0.05, ** *p* < 0.01, *** *p* < 0.001 vs. DMSO group. Error bars represent the standard deviations of the results from three independent experiments. DEA: Dehydroeburicoic acid; FP: fluorescence polarization; DMSO: Dimethyl sulfoxide; GSK3β: Glycogen synthase kinase 3β; IgG: Immunoglobulin G; Keap1: Kelch-like erythroid cell–derived protein with CNC homology–associated protein 1; Nrf2: Nuclear respiratory factor-2; MTT: (3-(4,5-dimethylthiazol-2-yl)-2,5-diphenyltetrazolium bromide) tetrazolium reduction.

The fluorescence polarization assay is a commonly used high-throughput assay to detect PPI inhibitors in solution. We used labeled Nrf2 peptides, bearing the high–affinity ETGE motif, which is recognized by Keap1, as fluorescent tracers to monitor for inhibition of the Keap1–Nrf2 interaction. ML334 is the first non–covalent small molecule inhibitor of Keap1–Nrf2 interaction reported and is distinct from other Nrf2 inducers [47]. In this paper, ML334 was used as a positive control. Compounds **1**–**13** from *A. cinnamomea* and ML334 were screened against the Keap1–Nrf2 interaction by FP (Figure 1A). The preliminary screening results showed that DEA (**1**) was the most potent compound, which disrupted the Keap1–Nrf2 interaction by 62% inhibition at 50 µM (cf. ML334: 60% inhibition). The NMR spectrum of DEA (**1**) is presented in Supplementary Figure S1. The potency of DEA (**1**) was further investigated in a dose-response assay, revealing an EC_50_ value of 14.1 ± 0.1 µM against Keap1–Nrf2 PPI (Figure 1B). We also explored the inhibitory effect of DEA (**1**) against GSK3β, which negatively regulates Nrf2 in a fashion independent of Keap1. 10 µM of DEA (**1**) reduced GSK3β activity by 58.9%, making it more potent than ML334 at the same concentration (11.3% inhibition) (Figure 1C). Moreover, a dose-response assay revealed an EC_50_ value of 8.0 ± 0.7 µM against GSK3β activity, indicating that DEA (**1**) is an effective GSK3β inhibitor (Figure 1D). In the nucleus, Nrf2 binds to the ARE to activate antioxidant gene transcription [14]. Thus, we tested the transcriptional activity of ARE after treatment of LO2 cells with DEA (**1**) and ML334 (10 µM) using the dual luciferase assay kit (Figure 1E). The activity of ARE was significantly increased by 54% in the treatment of DEA (**1**) compared to 27% in the ML334 group. The results showed that DEA (**1**) notably activates the transcriptional activity of ARE compared to ML334 in cellulo.

### 2.2. DEA (**1**) Exhibits Low Cytotoxicity In Cellulo

The cytotoxicity of compound DEA (**1**) was detected in both human normal cell lines LO2 and HEK 293T, and the hepatocellular carcinoma cell line HepG2. After treatment with different concentrations of DEA (**1**), no significant cytotoxicity was observed (Figure 1F–H). These data indicate that DEA (**1**) could be potentially safe for treating ALD in vivo.

### 2.3. DEA (**1**) as a Dual Inhibitor of Keap1 and GSK3β

**Figure 2:**
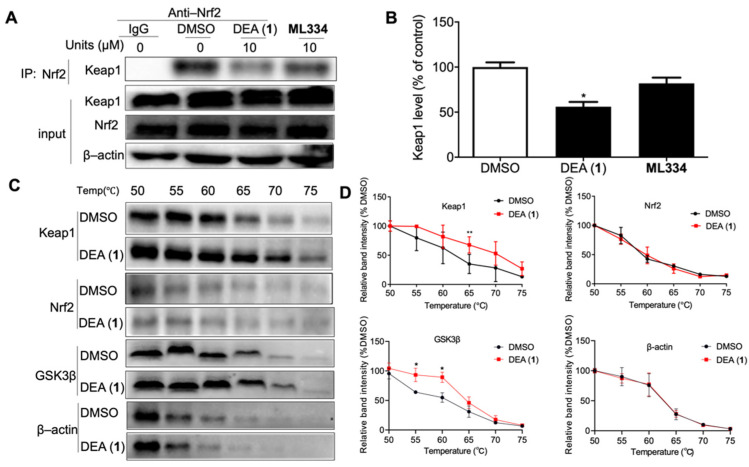
Compound DEA (**1**) releases Nrf2 by binding with Keap1 and GSK3β. (**A**) LO2 cells were treated with 10 µM DEA (**1**) and positive control ML334 for 8 h. Interactions between Keap1 and Nrf2 in LO2 cells were examined by WB and Co–IP. (**B**) Quantification analysis of Keap1 in WB. Error bars represent the standard deviations of the results from three independent experiments. (**C**) LO2 lysates were treated with 10 µM DEA (**1**) or DMSO and incubated at room temperature for 30 minutes. Then, CETSA was performed to assess Keap1, Nrf2, GSK3β, and β–actin thermal stability. (**D**) Quantification analysis of Keap1, Nrf2, GSK3β, and β–actin in WB. Data are represented as mean ± SD. * *p* < 0.05, ** *p* < 0.01 vs. DMSO group (Student’s *t*-test). Error bars represent the standard deviations of the results from three independent experiments. Anti–Nrf2: anti–Nrf2 antibody; WB: Western blot; Co–IP: co–immunoprecipitation.

The ability of DEA (**1**) to disrupt the Keap1–Nrf2 binding in cellulo was monitored through a Co–IP experiment using human normal liver cell line LO2 cells (Figure 2A). After treatment with DEA (**1**) (10 µM) or ML334 (10 µM) for 8 h, there was a 45% reduction of Keap1 co-precipitated with Nrf2 for DEA (**1**) as compared to 12% inhibition for ML334, indicating that DEA (**1**) could more effectively inhibit the Keap1–Nrf2 interaction compared to ML334 in living cells (Figure 2B).

We investigated the ability of DEA (**1**) to target Keap1, Nrf2, and GSK3β in the cellular environment using CETSA. LO2 cell lysates were incubated with 10 µM of DEA (**1**) at room temperature for 30 min. Then, aliquots were heated individually at different temperatures and the protein in the soluble fraction was quantified by WB. Compound DEA (**1**) significantly stabilized Keap1 (*ΔT_m_*: ca. 4.5 °C) and GSK3β (*ΔT_m_*: ca. 3.9 °C), while having no observable effect on the thermal stability of Nrf2 and β–actin (Figure 2C,D). This result suggests that DEA (**1**) can bind with Keap1 and GSK3β, even within the complicated environment of cell lysates.

### 2.4. DEA (**1**) Targets Keap1 and GSK3β to Induce Nrf2 Accumulation in the Nucleus and the Expression of Downstream Antioxidant Proteins

**Figure 3:**
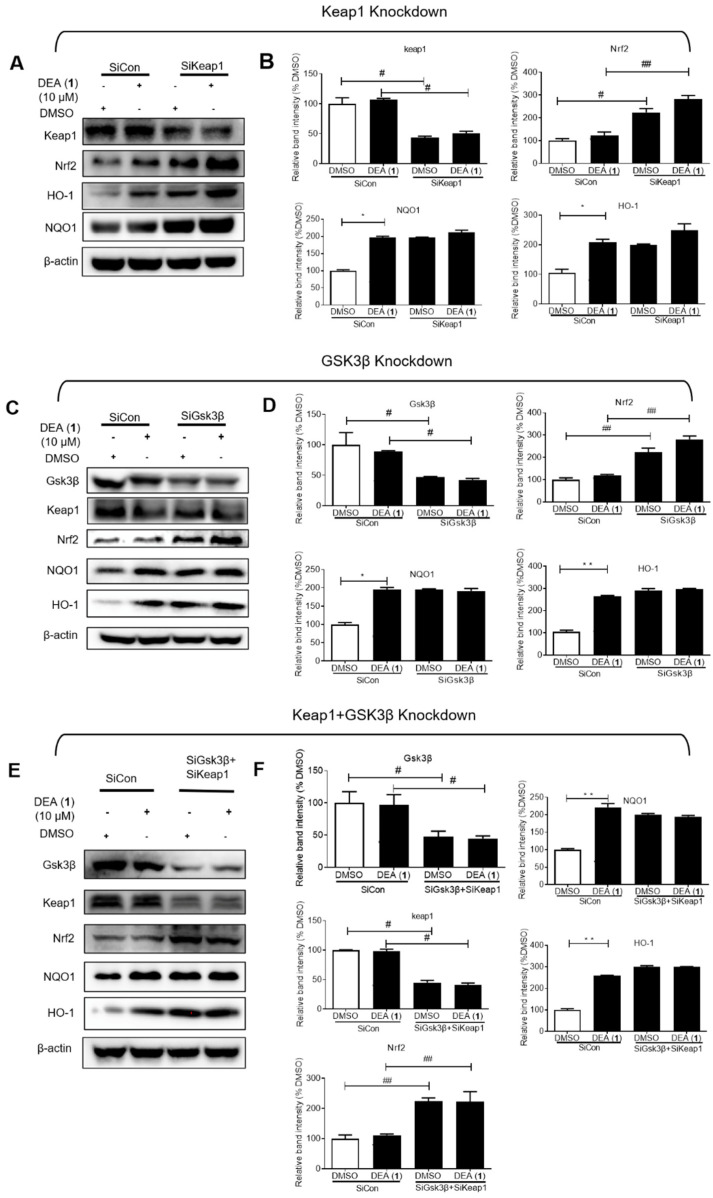
Compound DEA (**1**) regulates the antioxidant effects, at least in part, by targeting Keap1 and GSK3β. (**A**) Keap1 knockdown decreased the antioxidant effect of DEA (**1**) in LO2 cells. Keap1, Nrf2, *HO*–*1*, and *NQO1* were blotted to control for total protein levels. (**B**) Quantification analysis of Keap1, Nrf2, *HO*–*1*, and *NQO1* levels in Keap1 knockdown cells. Data are represented as mean ± SD. ^#^ *p* < 0.05, ^##^ *p* < 0.01 vs. control group, * *p* < 0.05 vs. DMSO. Error bars represent the standard deviations of the results from three independent experiments. (**C**) GSK3β knockdown decreased the antioxidant effect of DEA (**1**) in LO2 cells. GSK3β, Nrf2, *HO*–*1*, and *NQO1* were blotted to control for total protein levels. (**D**) Quantification analysis of GSK3β, Nrf2, *HO*–*1*, and *NQO1* levels in GSK3β knockdown cells. Data are represented as mean ± SD. ^#^ *p* < 0.05, ^##^ *p* < 0.01 vs. control group, * *p* < 0.05, ** *p* < 0.01 vs. DMSO group. Error bars represent the standard deviations of the results from three independent experiments. (**E**) Keap1 and GSK3β knockdown decreased the antioxidant effect of DEA (**1**) in LO2 cells. GSK3β, Nrf2, *HO*–*1*, and *NQO1* were blotted to control for total protein levels. (**F**) Quantification analysis of GSK3β, Keap1, Nrf2, *HO*–*1*, and *NQO1* levels in Keap1 and GSK3β knockdown cells. Data are represented as mean ± SD. ^#^ *p* < 0.05, ^##^ *p* < 0.01 vs. control group, ** *p* < 0.01 vs. DMSO group. Error bars represent the standard deviations of the results from three independent experiments. SiRNA Control: SiCon; SiRNA Keap1: SiKeap1; SiRNA GSK3β: SiGSK3β; SiGSK3β + SiKeap1: SiRNA GSK3β + SiRNA Keap1.

To verify whether Keap1 or GSK3β are direct targets of DEA (**1**), knockdown assays were performed. *NQO1* and *HO*–*1* are target antioxidant genes of Nrf2 that increase cytoprotection against oxidative stress [48] As expected, Keap1 knockdown in LO2 cells induced a noticeable rise of the expression levels of both Nrf2 and its gene targets, *NQO1* and *HO*–*1* (Figure 3A,B). However, treating Keap1 knockdown cells with DEA (**1**) led to no further rises in the *NQO1* and *HO*–*1* level, in contrast to the control cells, where DEA (**1**) treatment produced noticeable increases of *NQO1* and *HO*–*1*. These results suggest that DEA (**1**) activates *NQO1* and *HO*–*1* through targeting Keap1. Similarly, GSK3β knockdown induced Nrf2 and downstream antioxidant protein expression but without influencing Keap1 protein level (Figure 3C,D). In addition, DEA (**1**) had a diminished effect on *NQO1* and *HO*–*1* level in GSK3β knockdown cells as compared to the control cells. Finally, we knocked down GSK3β and Keap1 at the same time to verify the dual binding mechanism of DEA (**1**) on GSK3β and Keap1 (Figure 3E,F). The results showed that treating the GSK3β and Keap1 knockdown cells with DEA (**1**) did not cause a further significant induction of *NQO1* and *HO*–*1* expression.

Evidence for the hypothesis that DEA (**1**) acts through targeting both Keap1 and GSK3β comes from considering the Nrf2 level. In either the Keap1 knockdown or GSK3β knockdown cells, DEA (**1**) treatment resulted in slight increases of Nrf2 expression relative to DMSO. However, in the double Keap1 and GSK3β knockdown cells, treatment with DEA (**1**) did not lead to increases of Nrf2 expression. These results suggest that DEA (**1**) treatment mimicked the effects of double knockdown in increasing Nrf2 expression. Therefore, DEA (**1**) exerts its antioxidant effects by targeting both Keap1 and GSK3β.

**Figure 4:**
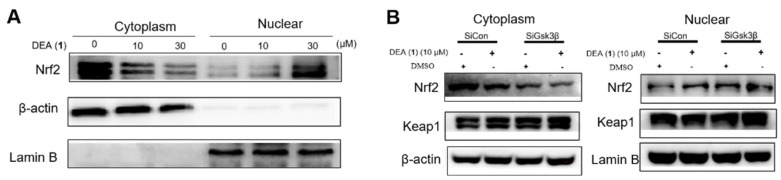
Compound DEA (**1**) increases Nrf2 translocation into the nucleus. (**A**) LO2 cells were treated with 10 or 30 µM compound DEA (**1**) for 8 h and then analyzed by WB to detect the level of Nrf2 in the nucleus and cytoplasm. β–actin and lamin B were used as internal controls. (**B**) GSK3β knockdown increased nuclear Nrf2 level. Keap1 and Nrf2 were blotted to control for the total protein levels. The data represent the standard deviations of the results from three independent experiments. SiRNA Control: SiCon; SiRNA GSK3β: SiGSK3β.

Inhibition of the Keap1–Nrf2 interaction is known to increase Nrf2 accumulation in the nucleus [47]. Meanwhile, GSK3β is also a negative regulator of Nrf2 accumulation by acting independently of Keap1 [49]. Therefore, we evaluated the Nrf2 level in the cytoplasm and nucleus of LO2 cells via WB after the treatment of cells with DEA (**1**) (10 µM) for 8 h. As shown in Figure 4A, DEA (**1**) significantly increased Nrf2 translocation into the nucleus in a dose-dependent manner. GSK3β knockdown also phenocopied compound DEA (**1**) treatment at inducing Nrf2 nuclear accumulation without influencing Keap1 level (Figure 4B). In summary, DEA (**1**) disrupts the Keap1–Nrf2 PPI and inhibits GSK3β to increase the translocation of liberated Nrf2 into the nucleus, thereby presumably increasing ARE transcriptional activity.

### 2.5. DEA (**1**) Activates Nrf2 Downstream Antioxidant Genes in ALD Model Cells

**Figure 5:**
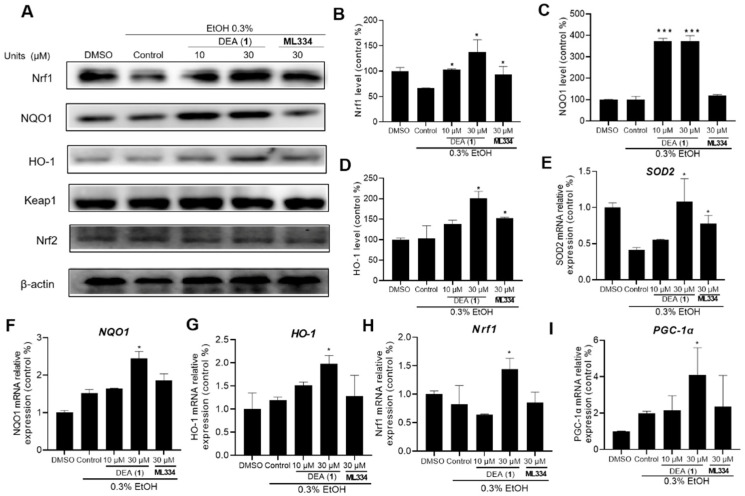
Compound DEA (**1**) induces antioxidant factor expression in the ALD cell model. (**A**) Effects of 10 µM, 30 µM DEA (**1**), and 30 µM ML334 on *Nrf1*, *NQO1*, *HO*–*1*, Keap1, and Nrf2 protein levels in LO2 cells after 8 h treatment. (**B**–**D**) Quantification analysis of *Nrf1* (**B**), *NQO1* (**C**), and *HO*–*1* (**D**) in WB. (**E**–**I**) Effects of DEA (**1**) and ML334 on (**E**) *SOD2*, (**F**) *NQO1*, (**G**) *HO*–*1*, (**H**) *Nrf1*, (**I**) *PGC*–*1α* mRNA levels in 0.3% EtOH–induced LO2 cells. Data are represented as mean ± SD. * *p* < 0.05, *** *p* < 0.001 vs. control group. The data represent the standard deviations of the results from three independent experiments. EtOH: Ethyl alcohol. *Nrf1*: Nuclear respiratory factor–1; *NQO1*: NAD(P)H dehydrogenase [quinone] 1; *PGC*–*1α*: Peroxisome proliferator–activated receptor γ coactivator 1α; SOD 2: Superoxide dismutase 2.

Nuclear Nrf2 heterodimerizes with musculoaponeurotic fibrosarcoma (Maf) to recognize ARE and promote the transcription of antioxidant genes, including *Nrf1*, *HO*–*1*, and *NQO1* [14]. Therefore, we evaluated the ability of DEA (**1**) to increase *Nrf1*, *NQO1*, and *HO*–*1* levels in ALD model cells, which were prepared by treating LO2 cells with 0.3% EtOH for 8 h. 0.3% EtOH was reported to induce oxidative stress in living cells [50,51,52]. In addition, a preliminary time-dependent assay was performed, which identified an optimal incubation time length of 8 h (Supplementary Figure S2). As shown in Figure 5A, DEA (**1**) (30 μM) induced *Nrf1* (Figure 5B), *NQO1* (Figure 5C) and *HO*–*1* (Figure 5D) protein levels by 1.2, 3.8 and 1.8-fold, respectively, in ALD model cells, making it more potent than ML334 (30 μM), which increased *Nrf1*, *NQO1*, and *HO*–*1* protein levels by 1.0, 1.1, and 1.5-fold, respectively.

Nrf2 directly controls the expression of the transcriptional factor *PGC*–*1α*, a regulator of mitochondrial biogenesis, while it also directly regulates the expression of mitochondrial antioxidant enzymes, such as superoxide dismutase 2 (*SOD2*) [53,54]. Hence, the effect of DEA (**1**) on mRNA levels of *SOD2*, *HO*–*1*, *NQO1*, *Nrf1*, and *PGC*–*1α* in 0.3% EtOH–treated LO2 cells was also evaluated. 30 μM of DEA (**1**) rescued *SOD2* mRNA level in ALD model cells by 2-fold, compared to 1.5-fold with ML334 (Figure 5E). DEA (**1**) (30 μM) also increased *NQO1* (Figure 5F) and *HO*–*1* (Figure 5G) mRNA levels by 2.0 and 2.5-fold, respectively, making it more potent than ML334 which increased *NQO1* and *HO*–*1* mRNA levels by 1.8 and 1.4-fold, respectively. In addition, the mRNA levels of *Nrf1* and *PGC*–*1α* were also increased 2-fold by compound DEA (**1**) (30 μM) (Figure 5H,I). The results also show a slight increase of *NQO1*, *HO*–*1*, and *PGC*–*1α* levels by 0.3% EtOH treatment alone, which has been previously observed in other studies [55]. However, the subsequent increases of antioxidant genes were much greater upon further treatment of DEA (**1**). Taken together, these results indicate that DEA (**1**) can activate Nrf2 downstream antioxidant genes in EtOH–treated human liver cells, a cellular model of ALD.

### 2.6. DEA (**1**) Repairs the EtOH Induced Mitochondrial Dysfunction and Improves the Antioxidant Activity in LO2 Cells

**Figure 6:**
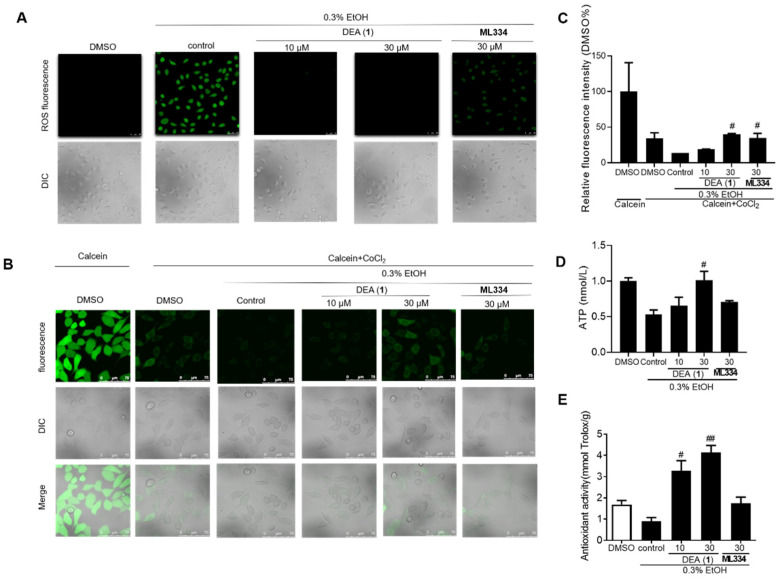
Compound DEA (**1**) protects mitochondrial dysfunction from 0.3% EtOH–induced injury. (**A**) 0.3% EtOH–induced LO2 cells were treated with 30 µM ML334, 10 or 30 µM compound DEA (**1**) for 8 h. Intracellular ROS level were detected by DCFH–DA probe and measured using the confocal laser scanning microscopy. (**B**) EtOH–induced LO2 cells were treated with 10 µM ML334, 10 or 30 µM compound DEA (**1**), DMSO for 8 h, and MPTP function was detected by fluorescence spectroscopy. (**C**) Quantification analysis of fluorescence in MPTP assay. (**D**) ATP level were detected using an ATP assay kit in 0.3% EtOH–induced LO2 cells. (**E**) Antioxidant activity of DEA (**1**) was detected in 0.3% EtOH–induced LO2 cells. Data are represented as mean ± SD. ^#^ *p* < 0.05, ^##^ *p* < 0.01, vs. control group. The data represent the standard deviations of the results from three independent experiments. ATP: Adenosine triphosphate; DCFH–DA: Dichlorodihydrofluorescein diacetate; MPTP: Mitochondrial Permeability Transition Pore Assay; ROS: Reactive oxygen species.

Inflammation and oxidative stress increase ROS production, and the activated Nrf2 was able to maintain low intracellular ROS level to protect the cell from oxidant injury [17]. Moreover, growing evidence shows that mitochondrial and oxidant injury induced by ethanol consumption play key roles in alcohol–induced liver injury [56]. Alcohol–induced ROS causes mitochondrial membrane depolarization and mitochondrial permeability transition (MPT), leading to hepatic apoptosis and necrosis [54]. Therefore, we detected the effect of DEA (**1**) and ML334 on ROS level in 0.3% EtOH–treated LO2 cells (Figure 6A). The results indicated that both **1** and ML334 could significantly decrease ROS level induced by EtOH. The MPTP assay showed that DEA (**1**) (30 μM) can restore MPTP function (40%) with a higher activity as compared to ML334 (34%) (Figure 6B,C). ATP is an indicator of mitochondrial function and ATP level are significantly reduced in ethanol–fed Nrf2^−/−^ mice [57]. DEA (**1**) (30 μM) rescued 50% of ATP in LO2 cells after EtOH treatment, making it more potent than ML334, which rescued 20% of the ATP level under the same conditions (Figure 6D). Finally, DEA (**1**) (30 μM) induced a higher increase of antioxidant activity (4-fold) compared to ML334 (2-fold) under the same conditions (Figure 6E).

## 3. Discussion

ALD is a serious chronic liver disease caused by oxidative stress and alcohol metabolism, which leads to massive global deaths [1]. Unfortunately, until now, there are no effective therapeutic drugs approved by the FDA for the treatment of ALD [58]. The pathogenesis of ALD is poorly characterized and research on the mechanism is still not clear. A number of reports have shown that the Keap1–Nrf2 pathway plays an important role in activating ARE antioxidant signaling [14]. Nrf2 is regulated via Keap1-independent pathways, including mitogen-activated protein kinase–Erk and PI3K–Akt pathways [59]. Among these regulatory pathways, GSK3β has emerged as a convergent point [60,61]. Because Nrf2 degradation and nuclear exclusion are regulated by GSK3β, it is critical in switching off the self–protective antioxidant stress response after injury [59,62]. Hence, targeting Keap1 and GSK3β to activate Nrf2 are potential therapeutic strategies for ALD.

Natural products provide diverse scaffolds with high bioactivity and low toxicity, and could be developed as promising candidates in drug development [63]. *A. cinnamomea* is a traditional Chinese herb that is a component of various medicines [64]. Triterpenoids are the major constituent in *A. cinnamomea*, and several studies have demonstrated their antioxidative and hepatoprotective effects [65,66]. However, the hepatoprotective mechanism of these compounds has not been extensively explored.

**Figure 7:**
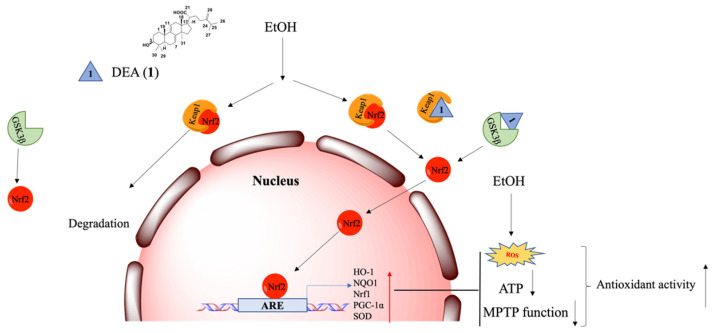
Hepatoprotective mechanism of the dual inhibitor DEA (**1**) against EtOH–induced cell injury.

DEA (**1**) has previously shown promising hepatoprotective activity in some studies [67], including in mouse models [39,67,68,69]. In recent years, many Keap1–Nrf2 PPI inhibitors and GSK3β inhibitors have been reported for various indications, such as Alzheimer’s disease, cancers, Parkinson’s disease, kidney diseases, Acetaminophen (APAP)–induced liver injury, and ferroptosis [30,70,71,72,73,74]. However, few have been studied in the context of ALD. In our present study, we discovered DEA (**1**) as a potent Keap1–Nrf2 PPI inhibitor from *A. cinnamomea* extracts. We explored the detailed mechanism of hepatoprotective action of DEA (**1**) in ALD, which acted via targeting both the Keap1–Nrf2 PPI and GSK3β (Figure 7). To our best knowledge, the dual inhibition mechanism of DEA (**1**) in ALD has not been reported before.

In in vitro assays, DEA (**1**) inhibited the Keap1–Nrf2 PPI (EC_50_ = 14.1 µM) as well as GSK3β kinase activity (EC_50_ = 8.0 ± 0.7 µM) with greater potency than the known Keap1–Nrf2 inhibitor, ML334. Cellular experiments confirmed the ability of DEA (**1**) to engage Keap1 and GSK3β in cellulo to activate Nrf2 and stimulate the expression of ARE–controlled genes. The specificity of DEA (**1**) was further confirmed using Keap1 and GSK3β knockdown experiments. DEA (**1**) treatment phenocopied dual knockdown of Keap1 and GSK3β, suggesting that DEA (**1**) acted simultaneously through both pathways to exert its biological effects. Moreover, DEA (**1**) increased Nrf2 nuclear accumulation, mimicking GSK3β knockdown, without influencing Keap1 level. Finally, we showed that DEA (**1**) could restore MPTP function and reduce ROS level induced by EtOH in ALD model cells. DEA (**1**) displayed low cytotoxicity in both normal and cancerous liver cells, indicating its potential safety for treating ALD in vivo. In summary, DEA (**1**) is a promising scaffold for the further development of dual inhibitors of the Keap1–Nrf2 PPI and GSK3β as therapeutic agents against ALD. The structural modification of compound DEA (**1**) is in progress and animal studies will be performed in due course.

## 4. Materials and Methods

### 4.1. Cell Lines and Culture

The human normal liver cell line (LO2), human embryonic kidney 293 T cell line, and HepG2 cell line were purchased from American Type Culture Collection (Manassas, VA, USA). Cells were cultured in Dulbecco’s modified Eagle’s medium (DMEM), with 10% FBS, 100 units/mL penicillin, 100 μg/mL streptomycin. Fetal bovine serum (FBS), DMEM, penicillin, and streptomycin were purchased from Gibco BRL (Gaithersburg, MD, USA).

### 4.2. Chemical

Compounds **1**–**13** (purity > 98% by HPLC analysis) were isolated from *A. cinnamomea* in a previous study [75]. New ^1^H and ^13^C nuclear magnetic resonance (NMR) were recorded for DEA (**1**) to verify its identity for this study (Supplementary Figure S1). Spectral data were consistent with our previous report [75].

### 4.3. Fluorescence Polarization Assay

The ability of compounds **1**–**13** and ML334 to inhibit Keap1–Nrf2 peptide binding was evaluated using a FP assay according to the manufacturer’s instruction (BPS Bioscience, San Diego, CA, USA). ML334 was a non–covalent small molecule inhibitor of the Keap1–Nrf2 and purchased from MedChemExpress (Princeton, NJ, USA). The tested compounds were dissolved in the provided buffer at the indicated concentrations. Then, the required volumes of Nrf2 peptide, Bovine Serum Albumin (BSA), Keap1 protein, and assay buffer were added to each well, followed by incubation for 30 min and the fluorescent polarization value was recorded.

### 4.4. Western Blot (WB) and Co–Immunoprecipitation (Co–IP)

The protocol was reported in a previous study [76]. In brief, LO2 cells were seeded at a density of 2 × 10^6^ cells in a six–well plate. LO2 lysate is harvested after incubation with 10 μM of compound DEA (**1**) or ML334 for 8 h. A 10% SDS–PAGE gel was used to separate the LO2 cell lysates, which were transferred to a PVDF membrane (Bio–Rad, Hercules, CA, USA). The membranes were incubated with antibodies anti–Keap1 (1:1000, Cell Signaling Technology, Danvers, MA, USA; Cat# 8047S), Nrf2 (1:1000, Proteintech, Wuhan, China; Cat# 16396-1-AP), GSK3β (1:1000, Cell Signaling Technology, Danvers, MA, USA; Cat# 9832S), *HO*–*1* (1:1000, Cell Signaling Technology, Danvers, MA, USA; Cat# 5853S), *NQO1* (1:1000, Cell Signaling Technology, Danvers, MA, USA; Cat# 3187S), β–actin (1:1000, Absin, Shanghai, China; Cat#: abs137975), or anti-lamin B (1:1000, Cell Signaling Technology, Danvers, MA, USA; Cat # 12586S) overnight. The membranes were washed with wash buffer three times. A secondary antibody (1:1000) was added to the membranes. After incubated for 2 h, the proteins bands were detected using enhanced chemiluminescent Plus reagents (GE Healthcare, Boston, MA, USA) and analyzed by Image Lab. The antibodies were purchased from Abcam (Waltham, MA, USA).

Co–IP between Keap1 and Nrf2 was performed following the protocol from Life Technologies. Briefly, lysates from 2 × 10^6^ LO2 cells were incubated with anti–Nrf2 or Rabbit mAb IgG (Abcam, Waltham, MA, USA; Cat#: ab205718) overnight. Then, samples were incubated with 10 μL Dynabeads (Life technologies, Foster, CA, USA) for 6 h. The Dynabeads were washed and analyzed by WB with the Keap1 antibody.

### 4.5. Real-Time Quantitative Polymerase Chain Reaction (RT–qPCR)

LO2 cells were incubated with 10 µM or 30 µM compound DEA (**1**) for 8 h and the total RNA was extracted using the NucleoSpin^®^ RNA Plus kit (Takara, Tokyo, Japan). We checked the RNA quality during the RT–qPCR operation and ensured that all the A260/280 value of samples were around 2, which is consistent with the literature [77]. We also performed an RT negative control and found no PCR amplification. Then, cDNA was synthesized using the PrimeScript™ RT Reagent Kit (Takara, Tokyo, Japan). The RT–qPCR conditions were as previously reported, with minor modifications [77]. DNA denaturation temperature: 95 °C (30 s); annealing temperature: 58 °C (30 s); extension temperature: 72 °C (45 s), number of cycles: 40. RT–qPCR was performed and analyzed as described previously [78]. The related primers are listed in Supplementary Table S1.

### 4.6. MTT Assay

The LO2, HUVEC and HEK 293 T cell lines were seeded at 1 × 10^4^ cells per well in 96–well plates and incubated with DMEM overnight at 37 °C in a humidified CO_2_ incubator to ensure attachment. The medium was then replaced with a medium that contained the different concentrations of compound for 48 h at 37 °C. The final concentration of DMSO was 0.1% or lower in all cases (including controls). The medium with 100 μL of MTT (3-(4,5-dimethylthiazol-2-yl)-2,5-tetrazolium bromide) reagent (1 mg/mL) was then replaced. After 4 h incubation, it was replaced with 100 μL of DMSO and measured using a microplate reader at 570 nm.

### 4.7. Detection of ROS and Antioxidant Activity

LO2 cells at a density of 2 × 10^6^ cells/well in a six-well plate were treated with different concentrations of DEA (**1**) or 10 μM ML334 for 8 h. ROS level and antioxidant activity were measured using the Reactive Oxygen Species Assay kit and the Total Antioxidant Capacity Assay Kit with FRAP, respectively, according to the manufacturer’s instructions (Beyotime, Shanghai, China).

### 4.8. Nuclear and Cytoplasmic Extraction

LO2 cells were incubated with 10 µM or 30 µM of DEA (**1**) for 8 h. The nuclear and cytoplasmic extraction followed the protocol from the manufacturer (Epigentek, New York, NY, USA, EpiQuik™ Nuclear Extraction Kit I, Catalog # OP-0002), Then, protein expression was analyzed by WB with the indicated antibodies.

### 4.9. GSK3β Kinase Assay

The ability of DEA (**1**) and ML334 to inhibit GSK3β activity was evaluated using luminescent kinase assay according to the manufacturer’s instruction (Promega Corporation, Madison, WI, USA). Briefly, the 384 well white plate was mixed with 1 μL of inhibitor, 2 μL of enzyme, and 2 μL of substrate/ATP mix and incubated at room temperature for 60 min. After 60 min, 5 μL of ADP–Glo™ reagent was added and incubated at room temperature for 40 min. Then 10 μL of kinase detection reagent was added, incubated at room temperature for 30 min and the luminescence value was recorded (integration time 0.5–1 s).

### 4.10. Dual Luciferase Reporter Gene Assay

LO2 cells were seeded in a six–well plate with 80% confluence in a DMEM medium for 24 h. The ARE–luciferase vector or a control plasmid with TurboFect transfection reagent (Thermo Fisher Scientific, Waltham, MA, USA) were transfected into LO2 cells according to the manufacturer’s instruction, and cells were incubated for 48 h. Then, LO2 cells were incubated with 10 µM compound DEA (**1**), 10 µM ML334 or DMSO for 4 h. The ARE activity was detected through the manufacturer’s instruction (Beyotime Dual Luciferase Reporter Gene Assay Kit, Shanghai, China).

### 4.11. Cellular Thermal Shift Assay (CETSA)

LO2 cell lysates were incubated with 10 μM compound DEA (**1**) and DMSO at room temperature for 30 min. The lysates are divided and heated individually at the indicated temperature. The supernatants of the heated lysates were collected after centrifugation, and then analyzed by WB with the Keap1, Nrf2, and β–actin antibodies.

### 4.12. Mitochondrial Permeability Transition Pore (MPTP) Assay

MPTP kit was used for evaluation of mitochondrial permeability transition according to the manufacturer’s instructions (Beyotime, Shanghai, China). A density of 1 × 10^4^ cells in a six–well plate, EtOH–induced LO2 cells were treated with 10 µM of ML334, 10 or 30 µM compound DEA (**1**), DMSO for 8 h, and then pretreated with calcein or calcein and CoCl_2_. Cells were stained by calcein acetoxymethyl ester (Calcein AM) and MPT function was detected by fluorescence spectroscopy.

### 4.13. SiRNA Gene Knockdown

The sequences of Keap1 siRNA are 5′-GGCCUUUGGCAUCAUGAACTT-3′ (sense) and 5′-GUUCAUGAUGCCAAAGGCCTG-3′ (antisense) [79]. GSK3β siRNA is 5′-GCAUUUAUCGUUAACCUAA-3′ (sense) and 5′-UUAGGUUAACGA UAAAUGC-3′ (antisense) [80]. Keap1 and GSK3β siRNA was transfected with Lipo3000 reagent (Thermo Fisher Scientific, Waltham, MA, USA) into 1 × 10^6^ LO2 cells following the manufacturer’s instruction. After incubation for 48 h, Keap1 and GSK3β protein expression and functional experiments were performed.

### 4.14. Statistical Analysis

Statistical significance was determined using the Student’s t-test for experiments comparing two groups. The data is homogeneous and follows a normal distribution before proceeding to the statistical analysis. Comparisons among groups were analysed using analysis of variance (ANOVA). All statistical tests were done using the GraphPad Prism version 8.0 software (GraphPad Software Inc. San Diego, CA, USA). All values were expressed as the mean ± standard deviation (SD). *p* < 0.05 means statistical significance.

## 5. Conclusions

Recent studies on ALD are scarce compared to research on other liver diseases, and there are still no highly satisfactory therapeutic options for ALD. To our knowledge, no dual inhibitor of the Keap1–Nrf2 PPI and GSK3β to treat alcohol-induced liver injury has been reported. This paper has revealed that DEA (**1**), one of the active triterpenes isolated from *A. cinnamomea*, could be a potential lead compound for treating ALD. Compound DEA (**1**) inhibited the Keap1–Nrf2 interaction and GSK3β activity both in vitro and in cellulo, and displayed low cytotoxicity at the concentrations required for inducing antioxidant activity. Moreover, DEA (**1**) could engage Keap1 and GSK3β in liver cells and increase Nrf2 nucleus translocation, thereby activating ARE transcriptional activity and upregulating antioxidant genes and mitochondrial biogenesis regulators. Significantly, compound DEA (**1**) displayed more potent hepatoprotective activity when compared with the classic Keap1–Nrf2 PPI inhibitor ML334. Therefore, the structure of DEA (**1**) may be optimized to expand the diversity, improve the activity, and be used in animal research. To our knowledge, this paper firstly reports DEA (**1**) acts as the dual inhibitor of Keap1–Nrf2 PPI and GSK3β with the potential for protecting against ALD. We anticipate that natural product DEA (**1**) can be considered as a potential scaffold for the development of clinical agents for treating ALD.

## Data Availability

Data is contained within the article and Supplementary Material.

## Supplementary Materials

The following supporting information can be downloaded at: https://www.mdpi.com/article/10.3390/ph16010014/s1, Figure S1: NMR data of dehydroeburicoic acid; Figure S2: The time dependent of ML334, compound 2, compound DEA (1) and induces antioxidant factor expression in the ALD cell model; Table S1: Primer sequences used for PCR analysis.
