# Supplementary material for: Dehydroeburicoic Acid, a Dual Inhibitor against Oxidative Stress in Alcoholic Liver Disease
Source: Pharmaceuticals (Basel). 2022 Dec 22;16(1):14. doi: 10.3390/ph16010014 (PMC9866905; doi:10.3390/ph16010014)
Supplement: Supplementary file 1 [file pharmaceuticals-16-00014-s001.zip › pharmaceuticals-2067590-supplementary.pdf]

Supplementary information

Dehydroeburicoic Acid, a Dual Inhibitor against Oxidative Stress in Alcoholic Liver Disease

Shasha Cheng [1,†], Yi Kuang [2,†], Guodong Li [1,3], Jia Wu [1], Chung-Nga Ko [4], Wanhe Wang [4,5], Dik-Lung Ma [4,*], Min Ye [2,*] and Chung-Hang Leung [1,3,6,*]

1 State Key Laboratory of Quality Research in Chinese Medicine, Institute of Chinese Medical Sciences, University of Macau, Macau SAR 999078, China
2 State Key Laboratory of Natural and Biomimetic Drugs, School of Pharmaceutical Sciences, Peking University, Beijing 100191, China
3 Zhuhai UM Science and Technology Research Institute, Zhuhai 519031, China
4 Department of Chemistry, Hong Kong Baptist University, Hong Kong SAR 999077, China
5 Institute of Medical Research, Northwestern Polytechnical University, Xi'an 710072, China
6 Department of Biomedical Sciences, Faculty of Health Sciences, University of Macau, Macau SAR 999078, China
* Correspondence: edmondma@hkbu.edu.hk (D.-L.M.); yemin@bjmu.edu.cn (M.Y.); duncanleung@um.edu.mo (C.-H.L.)
† These authors contributed equally to this work.

Table of Contents

Supplementary file S1 NMR data of dehydroeburicoic acid……………………...…………….2

Supplementary Figure S1. NMR spectra of dehydroeburicoic acid……………………………………...3

Supplementary Figure S2. The time dependent of ML334, compound **2**, compound DEA (**1**) and induces antioxidant factor expression in the ALD cell model………………………………………………………………………………………...4

Supplementary Table S1. Primer sequences used for PCR analysis……………….…….……4

Supplementary file S1

NMR data of dehydroeburicoic acid

Dehydroeburicoic acid: ^{1}H NMR (400 MHz, pyridine-d5) δ: 3.46 (1H, t, J = 7.9 Hz, H-3), 5.63 (1H, s, H-7), 5.39 (1H, d, J = 5.1 Hz, H-11), 1.09 (3H, m, H-18), 1.09 (3H, m, H-19), 1.03 (6H, m, H-26, 27), 4.91 (1H, s, H-28a), 4.95 (1H, s, H-28b), 1.23 (3H, s, H-29), 1.15 (3H, m, H-30), 1.03 (3H, m, H-31). ^{13}C NMR (100 MHz, pyridine-d5) δ: 36.7 (C-1), 29.1 (C-2), 78.4 (C-3), 39.7 (C-4), 50.1 (C-5), 23.9 (C-6), 121.7 (C-7), 143.2 (C-8), 147.0 (C-9), 38.2 (C-10), 117.0 (C-11), 36.4 (C-12), 44.7 (C-13), 50.9 (C-14), 32.0 (C-15), 27.7 (C-16), 48.5(C-17), 16.6 (C-18), 23.4 (C-19), 49.5 (C-20), 178.9 (C-21), 32.1 (C-22), 33.1 (C-23), 156.2 (C-24), 34.6 (C-25), 22.4 (C-26), 22.3 (C-27), 107.5 (C-28), 26.3 (C-29), 29.2 (C-30), 17.0 (C-31)

Supplementary file S2

Supplementary material Figure S1. ^{1}H and ^{13}C NMR spectra of dehydroeburicoic acid.

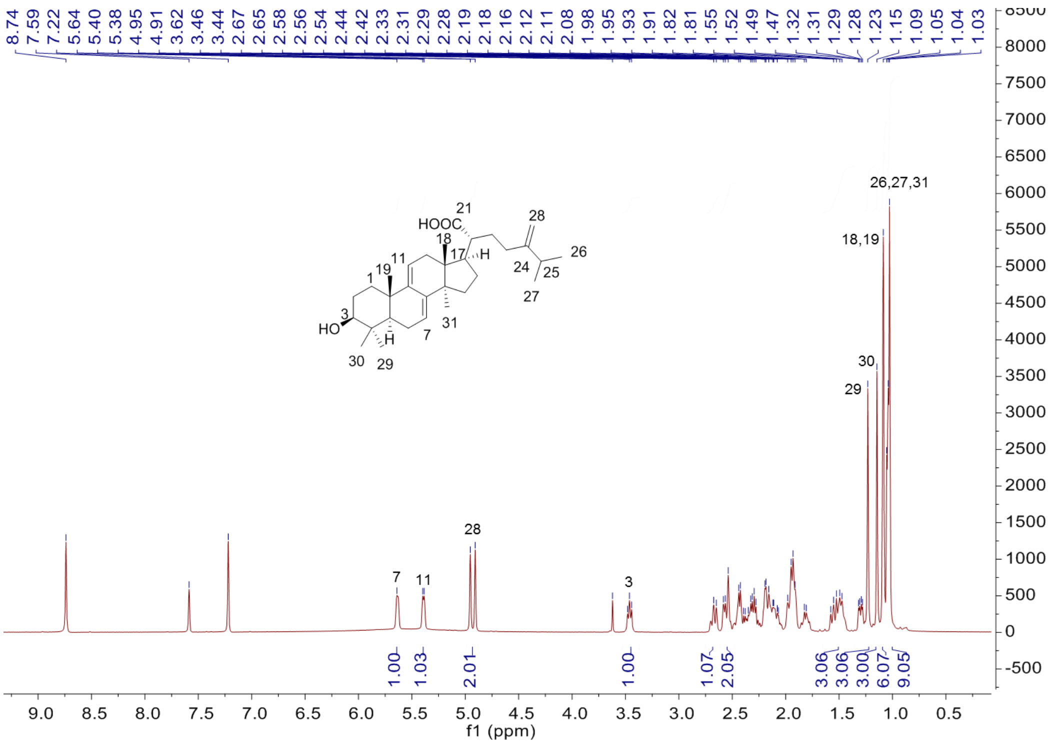

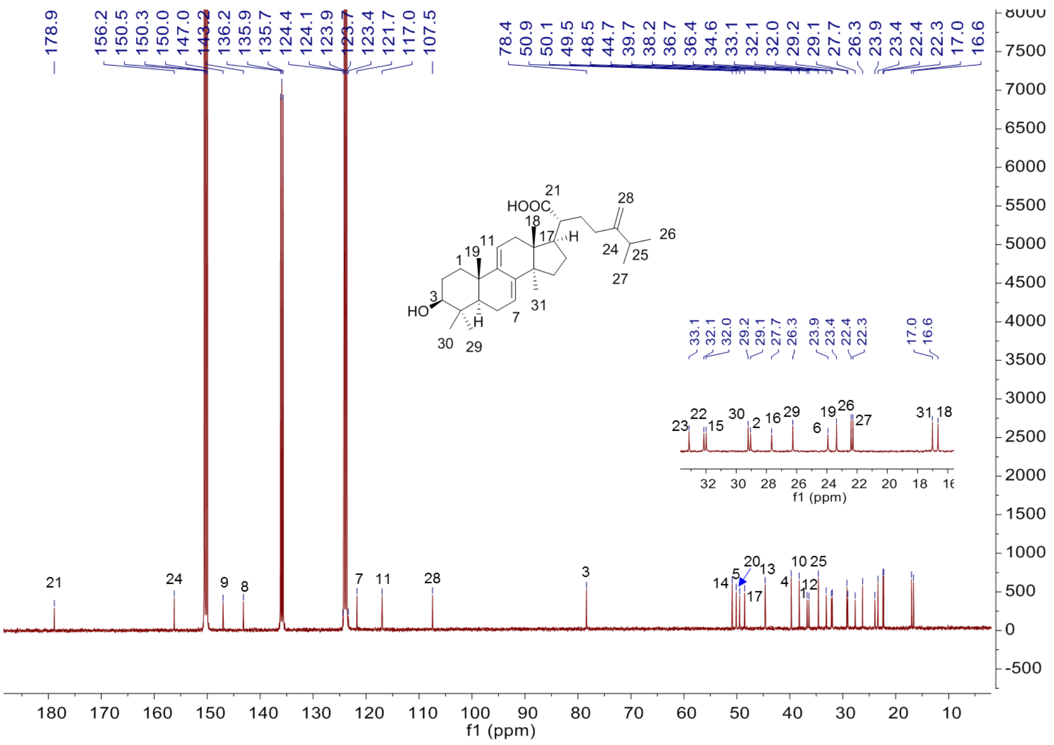

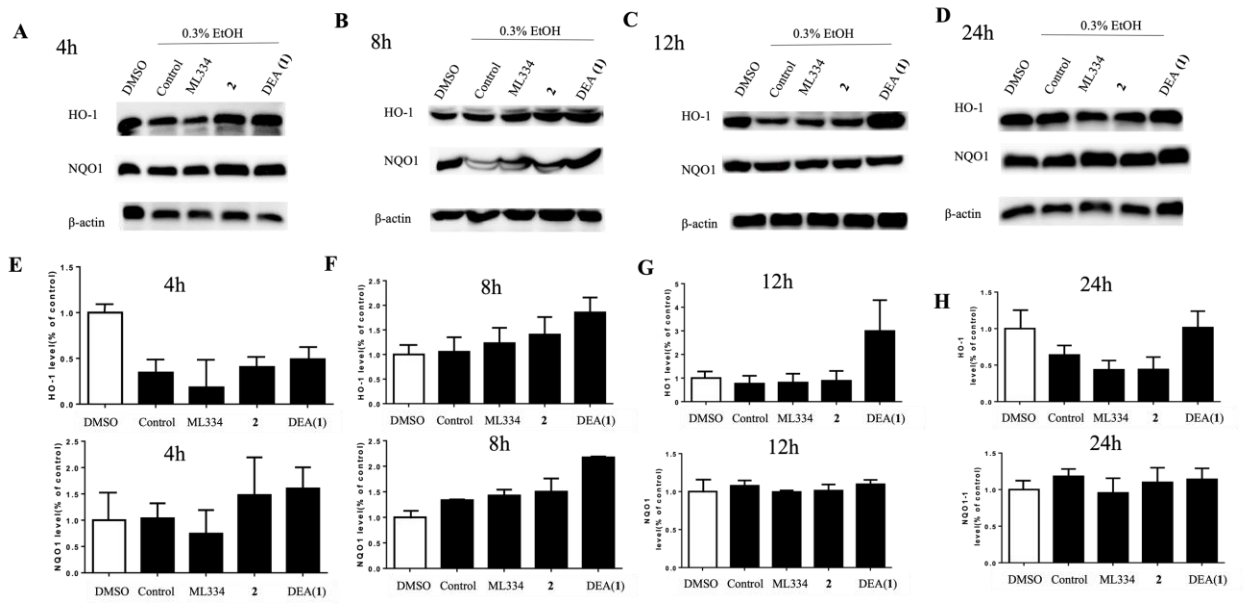

Supplementary Figure S2. The time dependent of ML334, compound **2**, and compound DEA (**1**) induces antioxidant factor expression in the ALD cell model. (A–D) Effects of 30 µM ML334, 30 µM of **2**, and DEA (**1**) on NQO1, HO–1 protein levels in LO2 cells after 4, 8, 12, 24 h treatment. (E–H) Quantification analysis of 4 h of HO–1 (upper) and NQO1 (lower) (E), 8 h of HO–1 (upper) and NQO1 (lower) (F), 12 h of HO-1 (upper) and NQO1 (lower) (G), 24 h of HO–1 (upper) and NQO1 (lower) (H) in WB.

Supplementary file S3

Supplementary material Table S1. Primer sequences used for PCR analysis.

RT-PCR primers	Forward primer	Reverse primer	Accession number	Amplicon size
β-actin	5'CATGTACGTTGCTATCCAGGC	5'CTCCTTAATGTCACGCACGAT	AK225414	250
NQO1	5'GAAGAGCACTGATCGTACTGGC	5'GGATACTGAAAGTTCGCAGGG	NG011504	312
HO1	5'GCCCTTCAGCATCCTCAGTTC	5'GTTTGAGACAGCTGCCACAT	NM002133	68
SOD2	5'CGTGACTTTGGTTCCTTTGAC	5'ATTTGTAAGTGTCCCCGTTCC	NG008729	116
Nrf1	5'CCAAGTGAATTATTCTGCCG	5'TGACTGCGCTGTCTGATATCC	KJ901609	494
PGC-1α	5′-GTCACCACCCAAATCCTTAT	5'ATCTACTGCCTGGAGACCTT	EU280319	131
